# A response to an inaccurate interpretation of the validity of the red category of the South African Triage Scale

**DOI:** 10.4102/safp.v62i1.5084

**Published:** 2020-03-02

**Authors:** Gareth D. Meyer, Tabitha N. Meyer, Charles B. Gaunt

**Affiliations:** 1 Department of Family Medicine and Rural Medicine, Faculty of Health Sciences, Walter Sisulu University, Mthatha, South Africa; 2 Department of Public Health, Faculty of Health Sciences, University of Liverpool, Liverpool, United Kingdom; 3 Directorate of Primary Health, Faculty of Health Sciences, University of Cape Town, Cape Town, South Africa

Dear editor,

We note with interest the article by Mohsen Ebrahimi and Amir Mirhaghi entitled ‘Red category criteria of the South African triage scale may need to be revised’ published in *South African Family Practice* on 19 March 2019. The article highlights some interesting points on over-triage when using the South African Triage Scale (SATS).

The article correctly notes that the result of over-triage is longer waiting times for true high-acuity patients. As a triage tool becomes more sensitive for high-acuity patients, it becomes less specific, resulting in higher over-triage rates. The practical result of this payoff is that in order not to have an acutely ill patient waiting for many hours in a green queue, some ‘well’ patients will end up in the red or orange queue. How well a given triage system balances sensitivity and specificity for true high-acuity patients is what determines its validity. The internationally recognised standard for over-triage set by the American Surgical Collaborative and Trial group aims at a rate of less than 50%, and, to our knowledge, the emergency medicine fraternity has not to date proposed any other target. In the referenced article by Meyer et al.,^1^ the reported over-triage rate (47.8%) was a composite high-acuity category comprising both red and orange patients to maintain comparability with previous studies. The over-triage rate of red-category patients alone was not reported. The red discriminators are almost certainly universally accepted emergencies, and if there are concerns about over-triage with the SATS, the orange category is more likely to be open to constructive criticism.

Ebrahim and Mirhagi also comment on Meyer et al.’s^1^ reported percentage of red-category patients seen within target time (49%) and state ‘it is possible that only half of red patients are correctly triaged’. While we agree that correctly triaged red patients should by their nature elicit immediate management, not seeing a patient within target times does not necessarily mean that the patient was not truly high acuity. While Ebrahim and Mirhagi imply in their article that not achieving target times is a marker of inappropriate assignation of red triage category to patients, we would argue that achievement of target times is a poor marker of true acuity because it is so heavily influenced by the availability of resources and patient load. As a practical example, if three high-acuity patients arrive simultaneously at an emergency department with one doctor and they are correctly designated as red using the SATS, the target of immediate management would not be achieved because of the non-availability of resources and not because of any shortcoming of the triage tool. No gold standard for true acuity can exist, and various markers including outcome, 30-day mortality, cost and expert opinion have been used as a proxy for true acuity against which to test validity. Waiting time has not been used as a marker of true acuity in any validity study that we are aware of.

In the referenced article by Meyer et al.,^1^ it is fundamental to note that the similarity between the over-triage rate in high acuity (red and orange patients) and the achievement of target times in red patients are coincidental, as no part of the study design aimed to link these two statistics, and any inferences made from it would be speculative at best.

Another point to note from the referenced article by Meyer et al.^1^ is that the times recorded pertained to when the doctor saw the patient and not necessarily when the patient received treatment. Triage selects for acuity not complexity. Nursing staff can competently manage many emergencies, such as hypoglycaemia and seizures, before the arrival of the doctor. We believe that this is a particular strength of the SATS in that it empowers nurses, who are often the first contact health care professional, to identify and treat emergencies in the emergency department.

Ebrahimi and Mirhagi make an excellent point that over-triage poses a risk for conflict between nurses and doctors. In our experience, we have found this to be the case when there is a lack of understanding amongst doctors that over-triage is not a mistake on the part of the nurse or a flaw in the triage system but rather an integral and necessary part of the calibration of a sufficiently sensitive triage system. For a nurse-led triage system to work, it is imperative that the doctors and nurses are well educated regarding the triage system, and strategies to encourage open dialogue should be implemented to deal with differences in opinion as and when they arise.

Another important point raised is the concern that the physiological Triage Early Warning Score (TEWS) may be overly sensitive in leading to assignation of Red triage category. It is well established that the TEWS without the discriminators is not sufficiently sensitive and results in unacceptably high levels of under-triage,^2,3,4^ and secondly, at present, all available data support the threshold of over seven to identify emergency patients.^5^

The article highlights some common, yet important, triage concerns and misconceptions, and we believe this letter provides some clarity.

Thank you

Dr T.N. Meyer

Dr G.D. Meyer

Dr C.B. Gaunt
